# Towards real-time cardiovascular magnetic resonance guided transarterial CoreValve implantation: in vivo evaluation in swine

**DOI:** 10.1186/1532-429X-14-21

**Published:** 2012-03-27

**Authors:** Philipp Kahlert, Nina Parohl, Juliane Albert, Lena Schäfer, Renate Reinhardt, Gernot M Kaiser, Ian McDougall, Brad Decker, Björn Plicht, Raimund Erbel, Holger Eggebrecht, Mark E Ladd, Harald H Quick

**Affiliations:** 1 Department of Cardiology, West-German Heart Center Essen, Essen University Hospital, University Duisburg-Essen, Hufelandstrasse 55, 45122 Essen, Germany; 2 Department of Diagnostic and Interventional Radiology and Neuroradiology, Essen University Hospital, University of Duisburg-Essen, Hufelandstrasse 55, 45122 Essen, Germany; 3 Department of General, Visceral and Transplantation Surgery, Essen University Hospital, University Duisburg-Essen, Hufelandstrasse 55, 45122 Essen, Germany; 4 Evasc Medical Systems, 107-1099 West 8th Avenue, Vancouver, BC V6H 1C3, Canada; 5 Institute of Medical Physics, Friedrich-Alexander-University Erlangen-Nürnberg, Henkestrasse 91, 91052 Erlangen, Germany

**Keywords:** Aortic stenosis, Transcatheter aortic valve implantation, Cardiovascular magnetic resonance, Real-time

## Abstract

**Background:**

Real-time cardiovascular magnetic resonance (rtCMR) is considered attractive for guiding TAVI. Owing to an unlimited scan plane orientation and an unsurpassed soft-tissue contrast with simultaneous device visualization, rtCMR is presumed to allow safe device navigation and to offer optimal orientation for precise axial positioning. We sought to evaluate the preclinical feasibility of rtCMR-guided transarterial aortic valve implatation (TAVI) using the nitinol-based Medtronic CoreValve bioprosthesis.

**Methods:**

rtCMR-guided transfemoral (n = 2) and transsubclavian (n = 6) TAVI was performed in 8 swine using the original CoreValve prosthesis and a modified, CMR-compatible delivery catheter without ferromagnetic components.

**Results:**

rtCMR using TrueFISP sequences provided reliable imaging guidance during TAVI, which was successful in 6 swine. One transfemoral attempt failed due to unsuccessful aortic arch passage and one pericardial tamponade with subsequent death occurred as a result of ventricular perforation by the device tip due to an operating error, this complication being detected without delay by rtCMR. rtCMR allowed for a detailed, simultaneous visualization of the delivery system with the mounted stent-valve and the surrounding anatomy, resulting in improved visualization during navigation through the vasculature, passage of the aortic valve, and during placement and deployment of the stent-valve. Post-interventional success could be confirmed using ECG-triggered time-resolved cine-TrueFISP and flow-sensitive phase-contrast sequences. Intended valve position was confirmed by ex-vivo histology.

**Conclusions:**

Our study shows that rtCMR-guided TAVI using the commercial CoreValve prosthesis in conjunction with a modified delivery system is feasible in swine, allowing improved procedural guidance including immediate detection of complications and direct functional assessment with reduction of radiation and omission of contrast media.

## Background

Transarterial aortic valve implantation (TAVI) has become a viable minimally-invasive treatment option for high-risk and non-surgical patients with severe symptomatic aortic stenosis [1]. Its remarkable clinical success has led to a revolutionary dissemination of this catheter-based treatment, which is currently performed in an ever-increasing number of patients with either the balloon-expandable Edwards Sapien (Edwards Lifesciences Inc., Irvine, CA, USA)[2] or the self-expandable Medtronic CoreValve (Medtronic, Inc., Minneapolis, MN, USA)[3] bioprosthesis, especially since Conformité Européenne approval. However, despite various advancements over the last years, TAVI remains a technically demanding interventional procedure including various challenging steps and a variety of potential procedural complications. A major procedural challenge during TAVI is to achieve exact valve positioning across the native aortic valve annulus, correct placement of the stent-valve being of paramount importance for procedural success and for patient outcome [4]. Ventricular embolization, coronary artery obstruction, or valve dislocation into the ascending aorta may lead to acute, potentially life-threatening complications, and even less severe malpositioning is associated with an increased rate of complications such as the occurrence of significant paravalvular regurgitation, which has been shown to negatively affect both immediate [5] and longer-term [3] outcome, or the need for novel pacemaker implantation [6] when the prosthesis is implanted too low.

Both procedural safety and valve positioning accuracy are influenced by the imaging modality used for procedural guidance. Currently, TAVI is guided by X-ray fluoroscopy and angiography. X-ray fluoroscopy and angiography, however, entail several shortcomings such as (a) radiation exposure, (b) limited 2D projection-like visualization with little soft-tissue contrast, which impairs immediate recognition of procedural complications (e.g. pericardial tamponade), and (c) the need for repeated injections of nephrotoxic contrast media to opacify the large-caliber aorta in order to determine the aortic annulus and the location of the coronary ostia during valve positioning and implantation. Although transoesophageal echocardiography including real-time 3D imaging may be used as a helpful adjunct imaging modality during TAVI [7], this technique has not eliminated the need for fluoroscopy and angiography and may itself cause complications. Moreover, it requires general anaesthesia instead of conscious sedation, which is desirable and increasingly used for TAVI. To overcome the limitations of conventional X-ray-fluoroscopy and angiography, guidance systems are currently under development that aim to facilitate the TAVI procedure for the X-ray operator [8-10]. C-arm computed tomography with automatic aorta segmentation and valve landmark detection represents such a technique that seeks to simplify implantation of the stent-valve by guiding valve positioning and deployment utilizing 3D overlay with the fluoroscopic images [9,10]. Nevertheless, such techniques still require fluoroscopy and angiography and do not offer a simultaneous, real-time acquisition of cardiovascular anatomy and the TAVI devices.

Cardiovascular magnetic resonance (CMR), on the other hand, offers real-time image acquisition with unrestricted scan plane orientation and unsurpassed soft-tissue contrast with simultaneous visualization of the interventional device and appears, therefore, potentially advantageous over X-ray fluoroscopy and angiography during device navigation through the vasculature, during passage of the native aortic valve, and during valve implantation. It permits on-line monitoring of cardiac function, immediate detection of vascular and cardiac damage, and real-time orientation for axial positioning and deployment of the prosthesis. Since all this may even be achieved without the need for nephrotoxic contrast media, not even gadolinium, real-time CMR (rtCMR) may also help to reduce the risk of postprocedural acute kidney injury, which has been shown to quadruple the risk of postoperative mortality in the elderly TAVI patient population who often present with pre-existing chronic kidney disease [11]. In addition to improved procedural guidance, CMR is useful for pre-interventional diagnostic evaluation of the aortic valve [12] and the vascular aortic anatomy for interventional planning, as well as for immediate evaluation of the result [13-15]. Thus, CMR may be envisioned as a single imaging modality for TAVI covering diagnostic, pre-interventional evaluation, procedural guidance, and post-interventional ascertainment of treatment success.

TAVI also appears particularly attractive for a CMR-guided approach, since device delivery is performed through large-diameter vessels (femoral or subclavian artery) and the delivery devices come with a rather large instrument caliber. Hence, TAVI is amenable to passive catheter tracking and may be performed without the need for additional, sophisticated device modifications as needed for active visualization approaches [16], making it easier to ensure CMR compatibility and safety. Our group has recently demonstrated in vitro that the commercially available, nitinol-based CoreValve bioprosthesis is suited for rtCMR-guided placement when using a modified and CMR-compatible delivery system, while the stainless steel-based Edwards Sapien stent-valve is not [17]. Based on these observations we pursued the idea of rtCMR-guided TAVI in vivo and sought to evaluate the preclinical feasibility of rtCMR-guided CoreValve implantation in swine as briefly reported in a recent research correspondence [18].

## Methods

### Animal preparation

In-vivo experiments were performed on 8 female domestic pigs weighing 70.5 to 86.5 kilograms. The experiments were conducted in approved animal facilities by an experienced and qualified (Federation of European Laboratory Animal Science Association certification) team including one attending veterinarian. All procedures were performed in accordance with current guidelines and legal requirements and were authorized by the regional governmental animal care and use committee (TSG G 1078/09; Duesseldorf, Germany). Anaesthesia was performed according to a well-proven protocol for CMR interventions in pigs [19]. In brief, the animals were sedated initially by intramuscular injection of ketamine (30 mg/kg), azaperone (2 mg/kg) and atropine (0.025 mg/kg), followed by an intravenous continuous infusion of propofol (2.29 mg/kg/h), midazolam (1.14 mg/kg/h) and fentanyl (0.009 mg/kg/h) for full anaesthesia. The pigs were intubated with an endotracheal tube and ventilated with 30% oxygen using a respirator (Oxylog 1000, Dräger Medical, Lübeck, Germany) with a ventilation rate of 10 to 12 breaths per minute. During the procedure, the animals were heparinized after sheath insertion and monitored with electrocardiogram (ECG) and oxygen saturation. After the experiment, the fully anaesthetized animals were euthanized by intravenous bolus injection of pentobarbital (80 mg/kg), as mandated by the experimental protocol and animal use guidelines.

### TAVI device and CMR-compatible guidewire

**Figure 1:**
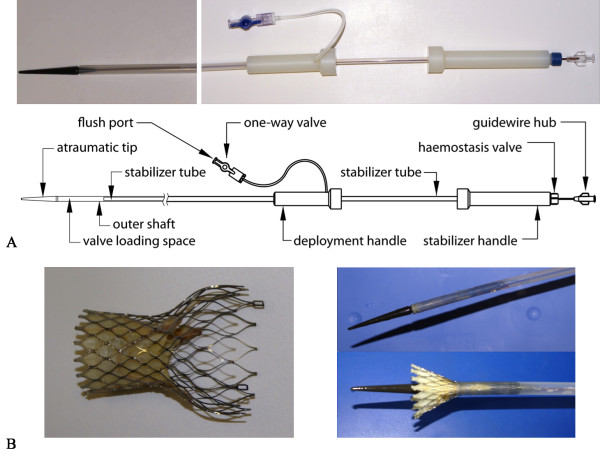
**The modified delivery device**. Photograph and schematic drawing of the modified, CMR-compatible delivery device for the CoreValve prosthesis with labeling of the relevant parts (A, reprinted from [17]). Photograph of both the expanded CoreValve prosthesis and the crimped stent-valve loaded in the polytetrafluoroethylene delivery catheter which offers stepwise valve deployment (B)

A recent, comprehensive in-vitro study by our group has demonstrated that the commercially available nitinol-based, self-expandable CoreValve bioprosthesis is potentially suited for rtCMR-guided placement using passive device visualization and real-time fast imaging with steady-state precession (TrueFISP) sequences [17]. While the commercial CoreValve prosthesis - providing artifact-free and detailed CMR-visualization and being already approved for safe CMR application in a static magnetic field of up to 3 Tesla - needed no modifications prior CMR application, its dedicated delivery system required effective modifications towards CMR compatibility and safety, which were performed with the help of Evasc Medical Systems (Evasc Medical Systems, Vancouver, BC, Canada). In summary, the modified delivery system was constructed with a polytetrafluoroethylene outer shaft and a nylon stabilizer tube without the use of any reinforcing ferromagnetic or electrically conducting wire braids or marker bands, which could potentially be object to radiofrequency heating in the CMR environment, but with preservation of rigidity and flexibility that resulted in a slight but practically negligible increase in the crossing profile from 18 to 20 French - when compared to the original CoreValve delivery catheter (Figure 1). For implantation, a deployment handle slides towards a stabilizer handle that is kept in a fixed position. This mechanism, which is common to other self-expandable devices (e.g. aortic stent grafts), allows for stepwise release of the prosthesis' tri-level nitinol stent-frame just like the original delivery system. Though not additionally endowed with the hinge and eyelet principle of the original device, the modified delivery device mechanically constrains the unsheathed part of the prosthesis safely until the first two thirds of the stent - the inflow part that anchors the stent-valve in the native annulus and the waisted mid-portion that guarantees unimpeded coronary artery perfusion - are expanded. This allows the operator to prevent stent displacement towards the left ventricle, which is commonly encountered during CoreValve implantation, by constantly pulling back the delivery system until the inflow part of the stent-valve is correctly anchored at the intended landing zone without accidentally releasing the prosthesis - similar to the original device. In-vitro, the modified, custom-built delivery-catheter provided excellent, artifact-free real-time visualization of catheter movement and valve deployment using fast TrueFISP sequences [17].

As for guidewires, which are currently a requirement for the TAVI procedure in vivo, we evaluated an CMR-compatible 0.035 inch polyetheretherketone-based prototype guidewire (Biotronik Vascular Intervention, Buelach, Switzerland) that has previously been used for (supra)aortic and iliac stenting as well as cava filter placement under rtCMRI guidance [20].

### Experimental protocol

In the sterile setting of a hybrid operative theatre with full anaesthesiological, surgical and angiographic equipment [21], a 22 F introducer sheath was surgically inserted into the common femoral artery for the transfemoral approach and into the subclavian artery for the transsubclavian approach. Angiography was performed to depict vascular and aortic valve anatomy, and delivery-device introduction and aortic valve passage were tested under fluoroscopic guidance. The obtained movies and still frames were digitally stored for later comparison of image quality and depicted information with CMR, but were not available during the subsequent CMR guided procedure. The delivery device was removed and the animals were transferred to the nearby CMR scanner room. After pre-interventional evaluation using standard CMR sequences, rtCMR guided TAVI was performed using TrueFISP sequences with subsequent CMR validation of procedural success. The pigs were then euthanized and intended valve positioning verified by ex-vivo histology.

### CMR scanner and sequences

Scanning was performed on a 1.5-Tesla whole-body CMR scanner (Magnetom Avanto, Siemens Healthcare Sector, Erlangen, Germany) equipped with a gradient system capable of a maximum amplitude of 40 mT/m and a slew rate of 200 T/m s^-1^. The animals were placed head first in a supine position inside the scanner on a multichannel spine phased-array radiofrequency (RF) coil with three 3-channel clusters of RF coil elements activated for signal reception (Tim technology, Siemens Healthcare Sector, Erlangen, Germany). Two 6-channel BodyFlex phased-array RF coils were placed anterior on the pig for signal reception. This setting has been previously used by our group to evaluate thoracic aortic stent-grafts for rtCMR-guided placement in vivo [22].

### Pre-interventional CMR evaluation

For pre-interventional preparation, vascular and cardiac anatomy was assessed using standard CMR sequences. Three-point localizer sequences were used to obtain optimal scan planes to depict the relevant anatomic cardiac and vascular landmarks. These scan planes were used for pre-interventional evaluation with steady-state free precession imaging with ECG gating and time-resolved retrospective image reconstruction (cine-TrueFISP retro) (imaging parameters: repetition time (TR) 40 ms, echo time (TE) 1,1 ms, flip angle 62°, field-of-view (FOV) 380 × 330 mm^2^, matrix 192 × 168, slice thickness 6 mm, bandwidth 930 Hz/pixel, image acquisition time (TA) 15 s for a single slice acquired over 20 phases of the cardiac R-R interval) and provided the basis for rtCMR guidance during subsequent implantation (see below). Flow-sensitive phase-contrast (PC) sequences were used to measure time-resolved aortic blood flow. ECG-triggered through-plane PC sequences and in-plane PC sequences in parasagittal orientation were used (imaging parameters: TR 62 ms, TE 3.5 ms, flip angle 30°, FOV 320 × 220 mm^2^, matrix 192 × 132, bandwidth 555 Hz/pixel, TA 1:52 minutes) with the velocity-encoded value (VENC) set to 100 cm/s on the basis of priorly acquired VENC scout sequences with VENCs ranging from 60 to 120 cm/s.

### Real-time CMR for interventional guidance

Interventional imaging guidance of the TAVI procedure was performed under rtCMR fluoroscopy. rtCMR fluoroscopy was based on a commercially available interactive real-time projection reconstruction TrueFISP sequence with radial k-space filling during free breathing and without cardiac triggering that was modified to achieve a frame rate of 7 frames per second (imaging parameters: TR 3.0 ms, TE 1.5 ms, flip angle 70°, FOV 360 × 360 mm^2^, matrix 192 × 192, bandwidth 1530 Hz/pixel, slice thickness 6 mm). Using sliding window reconstruction, forty-nine echoes were acquired for each new reconstructed image, resulting in real-time images with a frame rate of seven reconstructed frames per second. Images were displayed without detectable image reconstruction delay (< 200 ms). The images were displayed inside the scanner room using an 18" interventional in-room console (Siemens Healthcare Sector, Erlangen, Germany) to provide real-time visualization for the operator (PK). Image slice position, orientation and contrast parameters could be changed and adapted interactively from inside the scanner room while the sequence was running. For implantation, image position was adjusted in parasagittal orientations as needed to display the abdominal and thoracic aorta, the aortic arch and the ascending aorta including aortic annulus, left ventricular outflow tract (LVOT), left ventricle and mitral valve, respectively.

### Post-interventional validation

Following TAVI, real-time TrueFISP, cine-TrueFISP retro and flow-sensitive PC sequences were used to validate procedural success. cine-TrueFISP imaging was used for anatomic confirmation of adequate valve placement across the native aortic annulus and in relation to the LVOT and the mitral valve. Moreover, TrueFISP images were used to evaluate for periinterventional complications such as impairment of ventricular function, mitral valve function and pericardial effusion. PC sequences were repeated after TAVI to confirm good systolic blood flow without diastolic regurgitation indicating valvular or paravalvular leakage. While ECG-triggered, velocity-encoded, in-plane phase-contrast sequences in parasagittal orientation were used for visual, qualitative assessment of transvalvular blood flow after CoreValve implantation, through-plane phase-contrast sequences were used for quantitative assessment. Thereto, PC flow-curves integrated over time were obtained approximately 1 cm below and 1 cm above the prosthesis. This "safety margin" was chosen in order to avoid susceptibility artifacts and field distortion artifacts that might impact correct assessment of velocity and flow in terms of over- or underestimation. After completion of the post-interventional validation and on-table-euthanzation, macroscopic examinations of the explanted hearts were performed for validation of CMR-findings.

### Statistical analysis

Continuous variables are presented as means and standard deviations. Agreement of measurements based on cine-TrueFISP retro images with measurements on prior angiographic images and with autopsy results was determined by comparing differences to zero by *t*-test. A p value less than 0.05 was considered statistical significant.

## Results

### Pre-interventional evaluation

**Figure 2:**
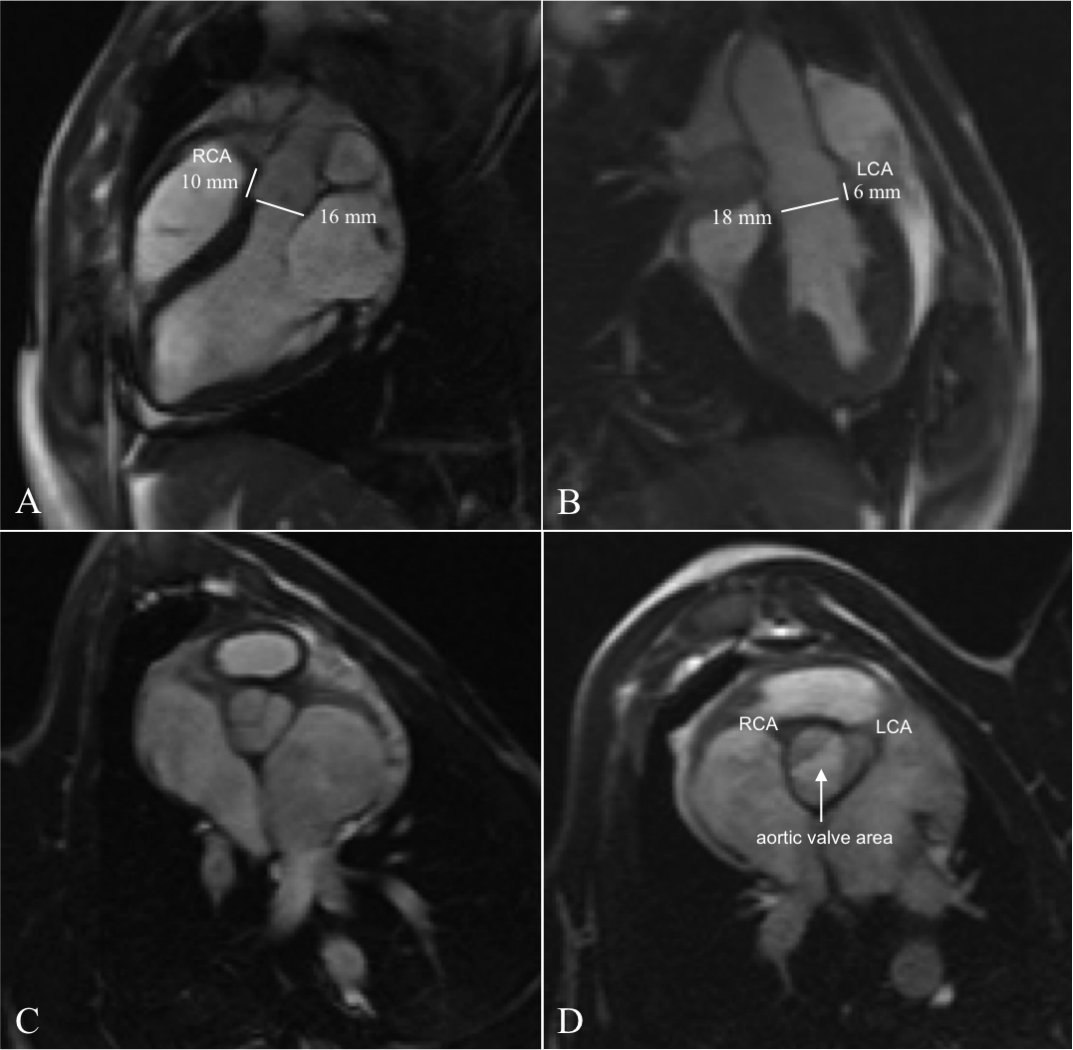
**High-resolution CMR images (cine-TrueFISP retro) of aortic valve and cardiac anatomy in parasagittal (A, B) and axial orientations (C, D) allowing precise, pre-interventional evaluation for rtCMR-guided TAVI**. The aortic annulus can be measured in the two orthogonal long-axis views (A, B), while aortic valve area can be clearly determined in the short-axis view during systole (D)

Three-point localizer sequences allowed for rapid detection (11 ± 3 minutes) and planning of all scan planes required for pre-interventional evaluation and for subsequent interventional guidance. Subsequent cine-TrueFISP retro sequences provided high-resolution, time-resolved images for a detailed assessment of the relevant cardiac and vascular structures with an acquisition time of 15 seconds (Figure 2). Measurements based on cine-TrueFISP retro images revealed an aortic annulus size of 17 ± 3 mm as measured in a sagittal, long-axis LVOT view which has the same orientation as the midoesophageal long-axis view on transoesophageal echocardiography that is routinely used for sizing measurements in clinical practice, an aortic arch diameter of 21 ± 4 mm and a distance of 9 ± 3 and 10 ± 2 mm from the aortic annulus to the left and right coronary ostium. Annulus measurements were in good accordance with measurements on prior angiographic images (mean error: 0.4 ± 0.3 mm, p = ns) and did not differ from autopsy results (mean error: 0.5 ± 0.2 mm, p = ns) demonstrating the accuracy of CMR. Access vessel size was 6.9 ± 1.2 mm and there were neither calcifications nor tortuosity impeding insertion of the 22 French access sheath and propagation of the delivery device.

Based on these measurements, the small CoreValve prosthesis (26 mm) was chosen for implantation in all pigs. Since the stent-frame - which is designed to be placed into the LVOT and across the aortic annulus with the sutured valve in supravalvular position - is designed for use in humans with annulus sizes between 20 and 23 mm, the prosthesis was, in effect, oversized for the animals. In addition, the ascending aorta of the pig was less elongated when compared to human anatomy and, thus, somewhat short for the 55 mm long prosthesis which is anchored there by its wide upper portion. These anatomical limitations of the pig model were accounted for during subsequent valve implantation. As a consequence, we intentionally aimed at various implantation depths of the proximal stent-frame within a target area between 4 and 20 mm below the aortic annulus, a range covering the usual implantation depth in humans [23]. Using the 8 mm diamond cell configuration for crude orientation, analog to human implantations, we started with "deep" implantations within this target area and gradually aimed at "higher" implantations over the course of our experiments to evaluate which depth would be optimal with respect to pig anatomy. Nevertheless, prerequisites for successful implantation were a functional placement of the CoreValve prosthesis across the aortic annulus without ventricular embolization, coronary artery obstruction or dislocation in the ascending aorta and maintenance of structural and functional integrity of the mitral valve.

### Real-time CMR for interventional guidance

**Figure 3:**
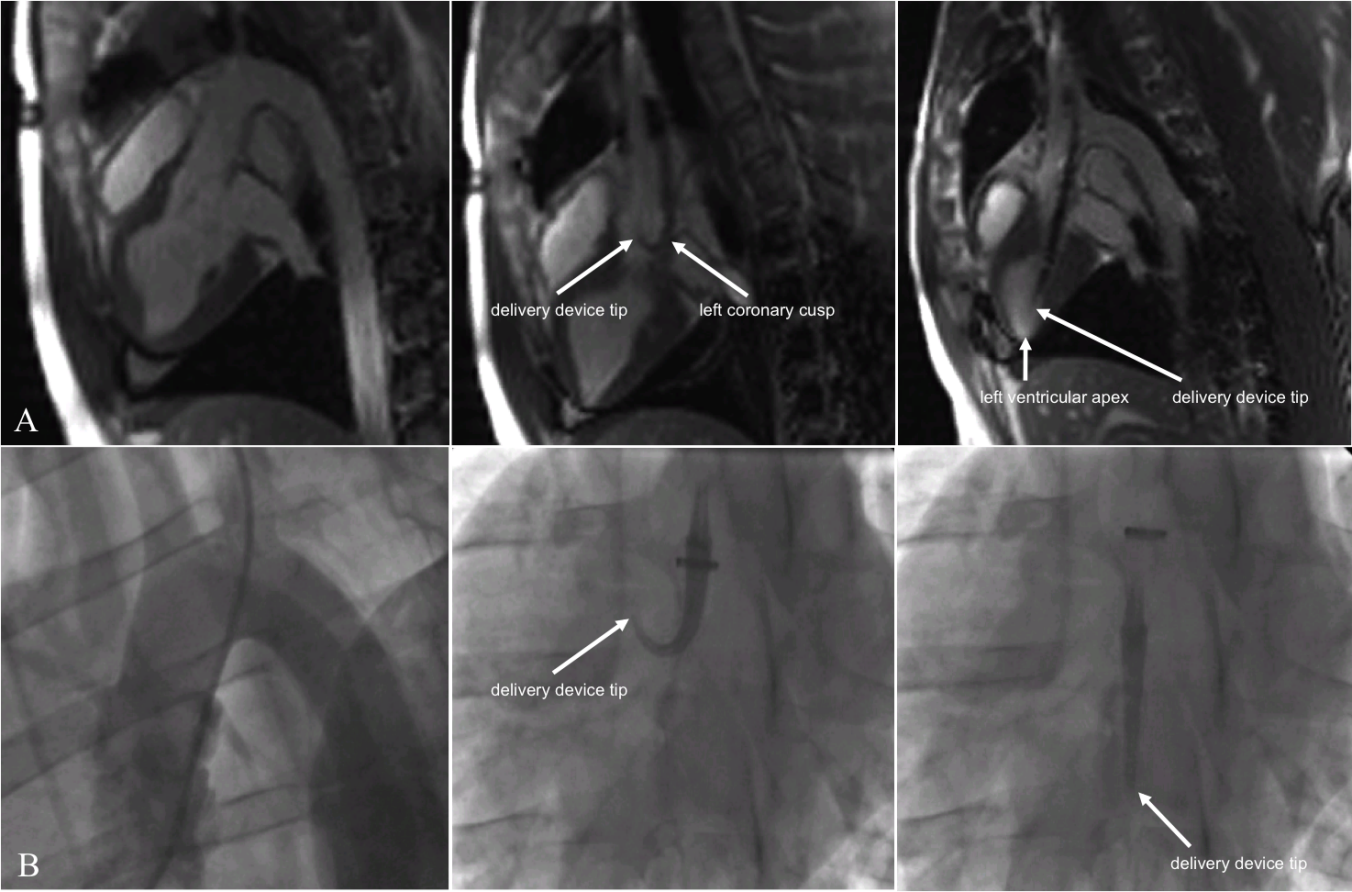
**Comparison of rtCMR (A) and fluoroscopic guidance (B) during aortic valve passage**. rtCMR provides real-time anatomic orientation with excellent soft-tissue contrast (A, left image). Fluoroscopy lacks this detailed soft-tissue contrast and requires (repeated) injection of contrast media to obtain a reliable orientation of the surrounding anatomy (B, left image). Moreover, rtCMR offers clear determination of the position of the delivery device and, especially, the delivery device tip similar to fluoroscopy (A and B, middle and right images). Note the bending of the delivery device tip in the middle image. With rtCMR it can be clearly determined that the delivery device is stuck in the left coronary cusp of the aortic valve and needs to be retracted and advanced again for safe valve passage. Once the valve is passed, rtCMR allows for precise advancement of the delivery catheter until the device tip is located in the left ventricular apex (A, right image)

rtCMR allowed for simultaneous visualization of the vasculature and the delivery system with the mounted stent-valve in good image quality suitable for interventional guidance. Mild susceptibility artifacts confined to the loaded stent-valve enabled adequate determination of the position of stent-valve and delivery system in relation to the surrounding anatomy without undue image distortion. This allowed for well-visualized propagation of the device through the vasculature superior to fluoroscopy which lacks the detailed soft-tissue contrast provided by CMR (Figure 3).

**Figure 4:**
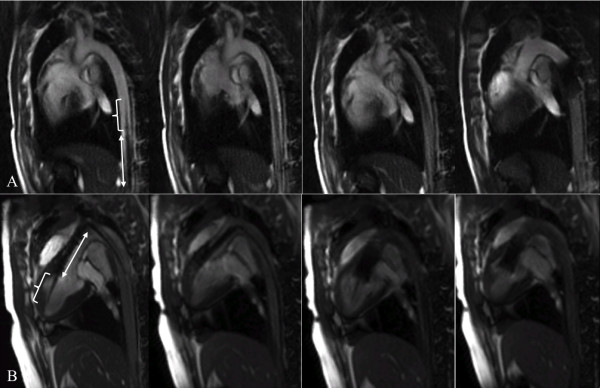
**Transfemoral TAVI under CMR guidance using real-time TrueFISP sequences**. Safe advancement of the delivery system with the mounted stent-valve up to the aortic arch which could not be passed in the first animal, resulting in controlled release of the prosthesis in the thoracic aorta distal to the left subclavian artery (A). Stepwise deployment of the CoreValve prosthesis across the native aortic annulus in the second transfemoral animal (B). Note the distinct susceptibility artifacts of the delivery system and the stent-valve, allowing precise determination of the position of the delivery device tip (bracket) and the mounted stent-valve (arrow)

In the first transfemoral animal, however, aortic arch passage remained unsuccessful. The delivery system with the mounted stent-valve could easily be advanced from the femoral artery access site through the thoraco-abdominal aorta, but the CMR-compatible guidewire - though well steerable and pushable - did not provide enough stiffness to allow passage of the rather steep aortic arch resulting in inability to correctly implant the valve which was finally deliberately deployed in the thoracic aorta distal to the left subclavian artery origin with perfect visualization (Figure 4A). Given the guidewire's insufficient stiffness for TAVI, we continued our experiments without the guidewire and focused on the subclavian approach, since it offers a more straight access route towards the aortic valve. Nevertheless, aortic arch passage was successful in the second transfemoral animal (Figure 4B).

**Figure 5:**
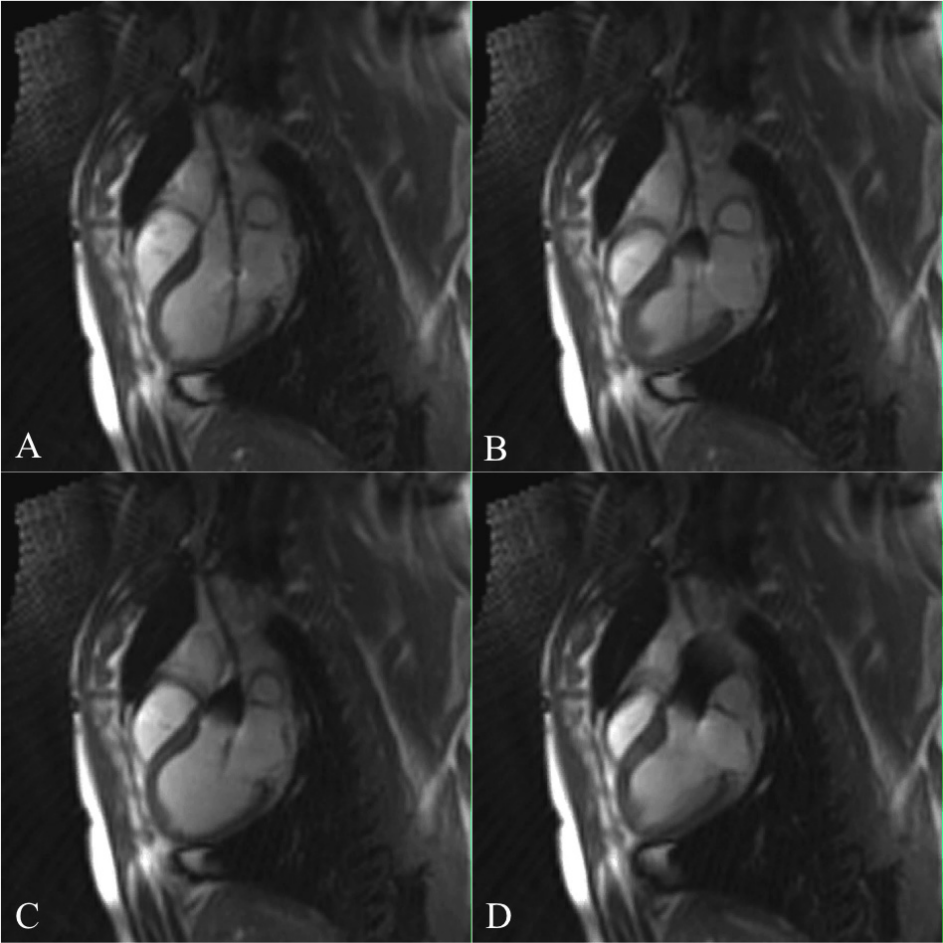
**Transsubclavian TAVI under CMR guidance using real-time TrueFISP sequences**. The CoreValve prosthesis was deployed stepwise across the aortic annulus under rtCMR monitoring using the commercially available interactive real-time projection reconstruction TrueFISP sequence with radial k-space filling during free breathing and without cardiac triggering. The sequence provides excellent instrument-to-background contrast and a high frame rate of 7 fps allowing for accurate positioning and for detailed monitoring of the valve deployment process.

In the following animals, rtCMR provided good visualization for navigation of the mounted stent-valve from the subclavian access site towards the aortic valve. In three animals, the device was initially advanced into the descending aorta. Due to the detailed soft-tissue contrast provided by CMR, this was immediately detected in real-time with good advantage over fluoroscopy and corrected. After successful steering into the ascending aorta, the aortic valve was carefully passed and the delivery devices were advanced until the device tips were located in the left ventricular apex. This maneuver was successful in all but one pig, in which the delivery device was inadvertently pushed with too much force too far into the left ventricle. This operating error caused ventricular perforation by the device tip with subsequent pericardial tamponade, which was detected without delay by rtCMR. The animal was euthanized when hemodynamic instability developed. In the other five pigs, rtCMR verified correct position of the delivery device tip in the left ventricular apex without signs of tissue damage. The susceptibility artifacts of the loaded stent-valve in combination with the simultaneously visible aortic annulus and mitral valve allowed for an adequate initial positioning of the prosthesis prior to release. Subsequently, the stent-valve was released stepwise with continuous adjustments of the position and successfully deployed under precise rtCMR visualization at the intended landing zone without dislocation (Figure 5). Thereto, a constant pull was exerted on the catheter system during deployment to compensate the typical movement of the stent-valve towards the left ventricle due to the tendency of the self-expandable stent-frame to occupy the largest space available. Lacking the excellent soft-tissue contrast of CMR, fluoroscopic guidance, for comparison, did not allow a real-time anatomic orientation during aortic valve passage and during positioning of the stent-valve, and required repeated contrast injections for adequate orientation (Figure 3).

After valve deployment, withdrawal of the delivery system was also adequately visualized under rtCMR. No other complications occurred. rtCMR acquisition time for instrument navigation, positioning and deployment of the prosthesis was 4 ± 2 minutes.

### Post-interventional validation

**Figure 6:**
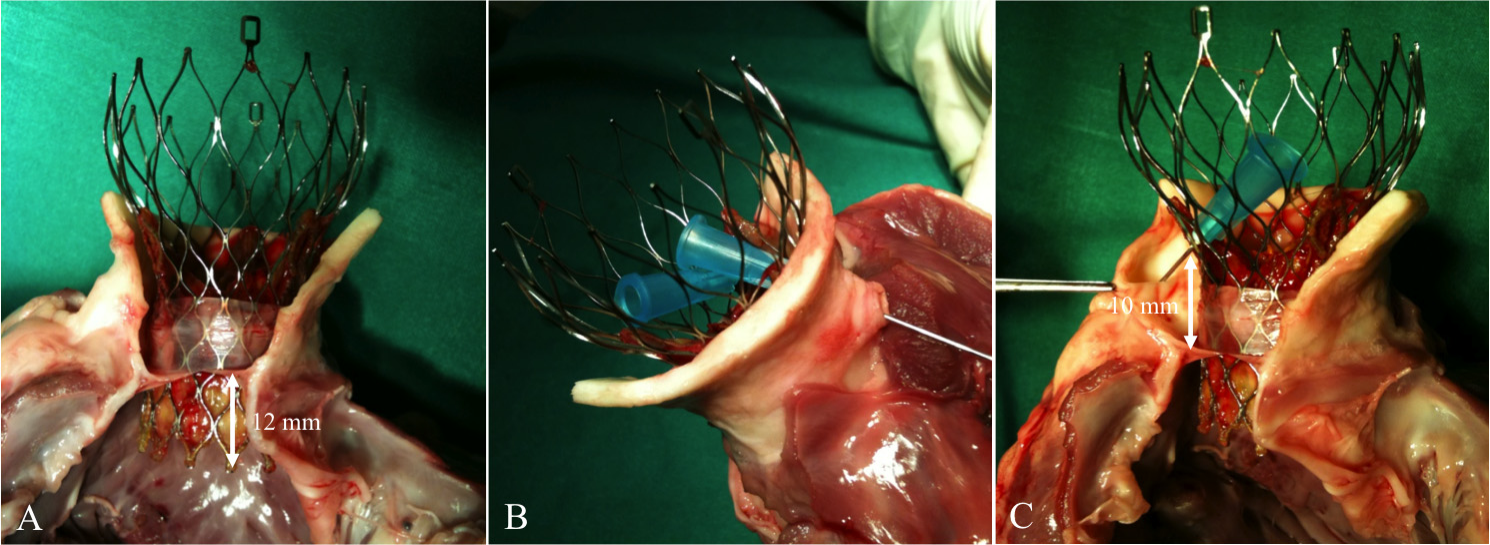
**Macroscopic examination of an explanted heart after rtCMR-guided TAVI**. The CoreValve prosthesis is correctly implanted across the aortic annulus with an implantation depth of approximately 12 mm into the LVOT (A). The waisted middle portion of the stent-valve is placed above the coronary ostia, thereby maintaining coronary artery perfusion as indicated by the probes (B, C)

**Figure 7:**
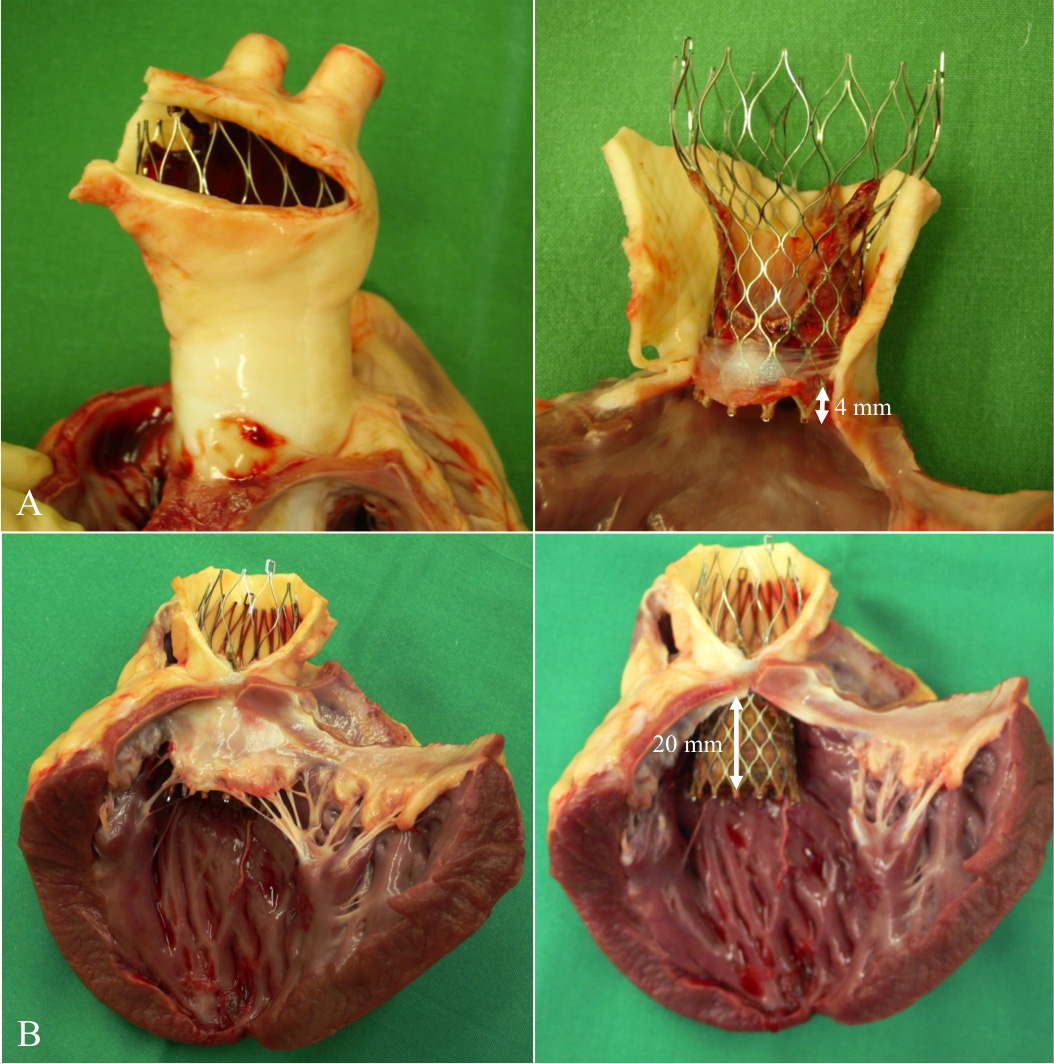
**Macroscopic images of a very high implantation across the aortic annulus with minimal implantation depth (A) and a deep implantation with an implantation depth of approximately 20 mm into the LVOT (B)**. While the high implantation resulted in a position of the upper part of the CoreValve stent-frame close to the supraaortic arteries due to the short ascending aorta of the pigs (A), the deep implantation demonstrated a close vicinity of the proximal stent-frame to the mitral valve (B)

**Figure 8:**
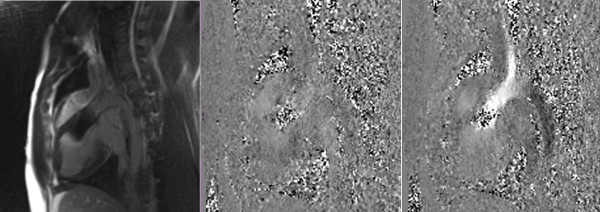
**Post-interventional flow-sensitive PC sequences in parasagittal orientation showing blood flow during diastole (left) and systole (middle)**. There is no turbulent flow during systole and also no regurgitant flow during diastole, qualitatively indicating the absence of relevant valvular and paravalvular regurgitation. Note that - due to the signal shielding effect - the position of the nitinol stent frame is depicted as a structure filled with image noise. For quantitative assessment, PC flow-curves integrated over time were obtained using through-plane PC sequences acquired in slices approximately 1 cm below and 1 cm above the prosthesis to avoid the influence of such field distortion effects on measurements.

Post-interventional CMR validation demonstrated a functional CoreValve bioprosthesis deployed across the aortic annulus within the intended target area between 4 and 20 mm in the LVOT and with maintenance of structural and functional integrity of the mitral valve and patency of the coronary ostia in all animals, as later additionally confirmed by macroscopic examination of the explanted hearts (Figure 6). Due to the relatively short ascending aorta in the pig model, a position approximately 10-15 mm in the LVOT provided the best anatomical results (Figure 6). A higher implantation - which may be considered optimal in humans - resulted in a position of the upper portion of the stent frame close to the insertion of the head vessels, while a deeper implantation in the left ventricular outflow tract lead to a close vicinity to the mitral valve, potentially impairing function, though this was not observed in our study (Figure 7). Flow-sensitive, ECG-triggered PC sequences confirmed good systolic transvalvular blood flow without diastolic regurgitation excluding valvular or paravalvular leakage in all implanted valves (Figure 8) as expected in view of the fact that an oversized prosthesis was used. The total procedure time was 61 ± 13 minutes.

## Discussion

The present study demonstrates the preclinical feasibility of entirely CMR-guided TAVI in a swine model. This was achieved using the commercially available CoreValve prosthesis without alterations and a modified, CMR-compatible delivery device. As a single imaging modality, CMR provided (a) comprehensive diagnostic evaluation of the relevant cardiac and vascular anatomy for adequate interventional planning, (b) reliable procedural guidance in real-time, (c) immediate evaluation of procedure-related complications, and (d) post-interventional validation of treatment success. The concept of rtCMR-guided TAVI was first described by Kuehne et al. in 2004. This archetype study in the preclinical, pioneering days of TAVI demonstrated the general technical feasibility and potential value of rtCMR guidance in acute animal experiments [24]. The authors implanted entirely custom-built self-expanding, nitinol-based stent-valves into the native aortic valves of seven pigs using passive device visualization. The stent-valves were loaded into a 10 French delivery catheter with two ferromagnetic markers and successfully implanted via the carotid and iliac artery. Subsequently, McVeigh et al. described the rtCMR-guided implantation of custom-built balloon-expandable stent-valves delivered by the surgical, transapical approach in Yorkshire swine [25]. They used conventional stentless aortic bioprostheses sutured into a commercial platinum-iridium stent and mounted onto a commercial balloon catheter. In their initial study, rtCMR-guided implantation with passive device visualization was technically successful in 6 out of 8 animals with 2 procedural deaths after valve deployment due to coronary artery obstruction and 2 additional procedural deaths during device manipulation. The same group further pursued the transapical approach focusing on chronic animal studies using their balloon-expandable valve [26] and, very recently, a custom-built self-expandable stent-valve for comparison [27]. Interestingly, the authors demonstrated shorter procedure times and easier manipulation with the self-expandable device, resulting in fewer procedural complications.

These custom-built, CMR-compatible devices provide excellent visualization for rtCMR guidance. Our study benefits from the use of the commercial, Conformité Européenne certified CoreValve bioprosthesis, which is already well-tested in the clinical arena with thousands of human implantations world-wide. The original CoreValve bioprosthesis in combination with a modified, CMR-compatible delivery device was chosen on the basis of a previous comprehensive in vitro evaluation of both currently commercially available stent-valves and their delivery catheters, which was recently performed by our group [17].

As shown by the present study, passive device visualization using real-time TrueFISP imaging with radial k-space filling provided excellent real-time visualization of the delivery system with the mounted CoreValve prosthesis and the surrounding anatomy during (a) navigation through the vasculature towards the aortic valve, (b) aortic valve passage, (c) initial positioning, (d) device deployment, and (e) catheter withdrawal. Such steady-state sequences, when used with high excitation flip angles, provide a high blood signal even without administration of a contrast agent and thus provide good instrument-to-background contrast in the CMR image [16]. The temporal resolution of seven reconstructed images per second, displayed without detectable image reconstruction delay on an in-room monitor, proved sufficient for direct and precise control of catheter movement. Moreover, rtCMR provided real-time monitoring of cardiac function and rapid detection of procedural complications.

In 6 of the 8 animals, a 26 mm CoreValve prosthesis was successfully placed across the native aortic annulus with implantation depths between 4 and 20 mm into the LVOT without ventricular embolization or dislocation into the ascending aorta, without coronary artery obstruction, and without structural or functional impairment of the mitral valve as confirmed by ex-vivo histology. Two implant failures occurred in our series. The first was a result of unsuccessful aortic arch passage and resulted in a controlled deployment of the stent-valve in the thoracic aorta distal to the left subclavian artery. Though considered an implant failure in this case, a controlled, safe deployment of the CoreValve prosthesis in the thoracic aorta without accidental vessel obstruction is sometimes required in clinical application when the CoreValve prosthesis dislocates into the ascending aorta during deployment and cannot be retrieved, as observed in approximately 10% of cases [4]. The second implant failure occurred due to perforation of the left ventricular apex by the delivery device tip as a result of an avoidable operating error, which was not related to periprocedural imaging guidance. However, this complication, which is observed in ~1% of cases [3], was detected by rtCMR without time delay and without the need for an additional imaging modality such as echocardiography. Both cases indicate that rtCMR might improve both precision and safety of the TAVI procedure.

In addition to improved procedural guidance, CMR provided a reliable pre-interventional diagnostic evaluation before as well as an adequate post-interventional validation after valve implantation. Our study could demonstrate that high-resolution TrueFISP retro sequences enable detailed visualization of all anatomic landmarks required for TAVI and allowed precise structural evaluation of the procedural result with good accordance with autopsy findings. It should be noted here, that CMR as a 3D imaging modality can also provide a comprehensive assessment of the elliptical shaped aortic annulus in different scan plane orientations. Such a detailed, 3D assessment might potentially have an impact on future recommendations for size selection of the stent-valve that is - in current clinical practice based - on a single 2D transoesophageal echocardiographic measurement in the midoesophageal, long-axis LVOT view. In this context, Koos et al. [28] have recently demonstrated in a cohort of TAVI patients that this single echocardiographic measurement correlates well with both CMR and dual-source computed tomographic measurements of sagittal aortic annulus diameters, the sagittal long-axis view on CMR and computed tomography basically having the same orientation as the midoesophageal long-axis view on echocardiography. In contrast, annulus diameters by transoesophageal echocardiography were significantly smaller than coronal aortic annulus diameters by CMR and by computed tomography. Regarding the TAVI strategy, the authors found a perfect agreement between transoesophageal echocardiography and sagittal CMR or computed tomography measurements. In contrast, decision-making based on coronal CMR or computed tomography measurements would have modified the TAVI strategy in 22% and 24% of cases when compared to the echocardiography-based strategy.

We could further demonstrate that functional assessment of the implanted stent-valves can be performed non-invasively using flow-sensitive PC sequences, which are already used for flow measurements in the clinical evaluation of valvular heart disease [29,30]. They allow detection of (para)valvular regurgitation immediately after valve implantation, which is important in clinical practice since hemodynamically relevant regurgitation after TAVI is associated with an increased in-hospital mortality [5] and may require immediate post-dilation of the prosthesis using a balloon or even valve-in-valve implantation. In our study, no relevant (para)valvular regurgitation was observed which may not be surprising in view of the fact that oversized prostheses were used.

Our study clearly shows the advantages of rtCMR-guided TAVI but also reveals its chief obstacle for translation into clinical application, namely the lack of suitable, CMR-compatible guidewires providing enough stiffness. When considering clinical application of rtCMR-guided TAVI, stiff guidewires are necessary. However, conventional metallic guidewires and guidewires with a nitinol core are not CMR-compatible since such long conducting structures might couple with the radiofrequency transmit energy of the body coil; this coupling could result in amplification of the local electric field and lead to excessive tissue heating, which presents a safety hazard precluding clinical application [31]. Currently, CMR-compatible guidewires are polyetheretherketone-based [20,32] or built from fiberglass [33] or other non-metallic, reinforced components and are passively visualized by their susceptibility artifacts, but do not provide mechanical stability equivalent to the commercial products used and required for TAVI (Amplatz Superstiff, Boston Scientific, MA, USA), as also shown in our study. Hence, the development of stiff guidewires suitable for CMR-guided TAVI is warranted. The development of CMR-compatible, metal-based guidewires might be an option. The precondition, however, remains that such guidewires do not act as radiofrequency antennas and must, consequently, include measures that counteract radiofrequency heating in the CMR environment.

When moving towards clinical application of rtCMR guided TAVI, other procedural and safety aspects should be taken into consideration and need to be discussed, specifically an adequate monitoring of heart rhythm and the issue of rapid right ventricular during preparatory balloon aortic valvuloplasty as well as an adequate procedural environment: A detailed ECG-monitoring is an essential prerequisite for TAVI. Arrhythmias (importantly ventricular tachycardia/fibrillation, bradycardia, atrial fibrillation), conduction abnormalities (importantly higher-degree AV-blocks, bundle-branch blocks) and signs of cardiac ischemia (importantly ST-elevation) need to be accurately assessable. The ECG-electrodes that have been used in our experimental setting were provided by the CMR scanner's manufacturer (Siemens Healthcare Sector, Erlangen, Germany) and were used for sequence triggering purposes only. With this setup, a comprehensive ECG-monitoring during a CMR-guided intervention is not possible. However, we did not observe any relevant decrease in heart rate/bradycardia suggestive of a higher-degree AV-block after TAVI. This might potentially be explained by the fact that implantation was performed into a juvenile, non-calcified, non-stenosed aortic valve. Nevertheless, a patient setting would require a CMR-compatible monitoring-equipment which is, however, already commercially available (e.g. Schiller MAGLIFE Serenity, Schiller Ottobrunn, Germany).

At present, balloon aortic valvuloplasty is generally performed as a preparatory step prior implantation of current transcatheter aortic valves. In order to prevent dislodgement of the inflated balloon into the ascending aorta as a result of cardiac contraction, rapid right-ventricular burst pacing is performed during valvuloplasty to induce a transient, functional cardiac arrest. Likewise, rapid right-ventricular pacing is needed for the implantation of the balloon-expandable Edwards prosthesis to ensure a stable position of the stent-valve during implantation. In contrast, the self-expandable CoreValve prosthesis does not require rapid pacing since its design allows for nearly continuous trans-aortic blood flow during stepwise implantation. Moreover, Grube and colleagues have recently demonstrated that CoreValve implantation is also feasible and safe without prior valvuloplasty [34]. Rapid right-ventricular pacing is, therefore, not required for CoreValve implantation and does not present another obstacle for procedural guidance by rtCMR.

Currently, TAVI is ideally performed in so-called hybrid operating rooms that offer a sterile environment with full angiographic, anaesthesiological and surgical equipment. Such rooms not only provide optimal conditions for the procedure but also provide a rapid interdisciplinary team approach for complication management. While peripheral vascular complications might already be managed by PTA and stent implantation under CMR-guidance, other complications might occur that cannot be addressed within the CMR scanner. Coronary artery obstruction, for example, may require percutaneous coronary intervention or even surgery. Hence, the CMR scanner should ideally be integrated in the architectural concept of such a hybrid room, which must warrant a rapid evacuation of patients from the CMR scanner in order to perform conventional X-ray guided intervention or surgery without delay. This can be ensured either by a swivelling operating table if the CMR scanner is integrated directly within the hybrid room or a rail-mounted operating table if the CMR scanner is accommodated in a separate, adjacent room. Alternatively, a patient transfer board on a non-magnetic patient transporter or even a movable CMR scanner might be considered, although these approaches are clearly more time-consuming.

As a limitation of our study, it also has to be noted that patients referred for TAVI usually show somewhat calcified and tortuous vessels. These vessels may present a more complex anatomy than seen in our animals. However, the benefits of the detailed soft-tissue contrast and 3D representation provided by CMR may be even more dramatic in these patients. Safe navigation through the tortuous vascular access routes is presumably simplified by rtCMR to guide operator adjustments and visualize device-related anatomic distortion. In view of the stenotic, calcified aortic valve in TAVI patients, potential CMR imaging artifacts arising from valvular and aortic root calcifications need to be acknowledged. Basically, calcifications appear as areas of low signal intensity or signal void on CMR imaging. Such signal voids can make edge discrimination of calcified valve leaflets difficult, especially during direct planimetry of aortic valve area in a cross-sectional view. They might also affect rtCMR-guidance of aortic valve passage during TAVI. However, in clinical practice, passage of the stenotic aortic valve is performed with a straight-tip guidewire in a probing fashion, since neither conventional X-ray angiography nor transoesophageal echocardiography allow precise, targeted steering of the wire through the small orifice. Hence, these imaging artifacts may be negligible at this stage of the procedure, and they should also not relevantly impair valve positioning and implantation since the areas of signal void are typically constraint to the areas of calcification and do not lead to further image distortion, which might impair detailed visualization of the relevant anatomical structures. Thus, the landing zone for the CoreValve prosthesis would still be accurately visible. However, these considerations remain speculative since a calcific aortic valve could not be simulated in the pig model. Interestingly, however, successful implantation of an oversized CoreValve prosthesis in a native, non-stenotic, non-calcified aortic valve without tissue damage and dislocation might potentially have clinical implications for future catheter-based treatment of aortic regurgitation which is currently not an indication for TAVI.

## Conclusions

The present in vivo experiments show that TAVI can be performed solely under rtCMR guidance. Using the commercially available CoreValve bioprosthesis and a modified, CMR-compatible delivery system which might potentially be transferred into clinical application without major additional effort, we could demonstrate that rtCMR with passive catheter tracking allowed for reliable monitoring of device navigation, valve positioning, and implantation across the native aortic annulus. In addition to immediate detection of procedure-related complications such as ventricular perforation and pericardial tamponade, CMR - as a single comprehensive imaging modality - provided a detailed pre-interventional evaluation of the vascular anatomy and of the annulus size as well as a direct functional assessment after valve implantation. Complimentary to reduction of radiation exposure and nephrotoxic contrast media, rtCMR guidance, therefore, provides advantages over conventional fluoroscopy and angiography and warrants further attention.

## Abbreviations

CMR: Cardiovascular magnetic resonance; cine-TrueFISP retro: Steady-state free precession imaging with ECG gating and retrospective image reconstruction; ECG: Electrocardiogram; FOV: Field-of-view; LVOT: Left ventricular outflow tract; PC: Phase-contrast; rtCMR: Real-time cardiovascular magnetic resonance; T: Tesla; TA: Acquisition time; TAVI: Transarterial aortic valve implantation; TE: Echo time; TR: Repetition time; TrueFISP: Fast imaging with steady-state free precession; VENC: Velocity-encoded value

## Competing interests

Philipp Kahlert was supported by an internal research grant from the University Duisburg-Essen (IFORES 10 + 2) and has received minor travel support from Edwards Lifesciences Inc. and Medtronic Inc. Holger Eggebrecht is a clinical proctor for Edwards Lifesciences Inc. and Medtronic Inc. and has received honoraria payment. Ian McDougall and Brad Decker are employees of Evasc Medical Systems, a division of evYsio Medical Devices ULC, Vancouver, Canada. The other authors have no financial interests in any products or companies described in this article. The investigated devices were provided by the companies free of charge.

## Authors' contributions

PK has conceived the study, acquired, analyzed and interpreted the data and drafted the manuscript. NP has acquired, analyzed and interpreted the data and has revised the manuscript for important intellectual content. JA has acquired, analyzed and interpreted the data and has revised the manuscript for important intellectual content. LS has acquired, analyzed and interpreted the data and has revised the manuscript for important intellectual content. RR has acquired, analyzed and interpreted the data and has revised the manuscript for important intellectual content. GK has acquired, analyzed and interpreted the data and has revised the manuscript for important intellectual content. IMD has made substantial contributions to the published study by designing the modified delivery catheter used in the experiments. BD has made substantial contributions to the published study by designing the modified delivery catheter used in the experiments. BP has made substantial intellectual contribution to conception and design of the study and has revised the manuscript for important intellectual content. HE has made substantial intellectual contribution to conception and design of the study and has revised the manuscript for important intellectual content. RE has made substantial intellectual contribution to conception and design of the study and has revised the manuscript for important intellectual content. MEL has made substantial intellectual contribution to conception and design of the study and has revised the manuscript for important intellectual content. HHQ has conceived the study, acquired, analyzed and interpreted the data and drafted the manuscript. All authors have read and given final approval of the manuscript.
